# Synthetic polyploid induction influences morphological, physiological, and photosynthetic characteristics in *Melissa officinalis* L.

**DOI:** 10.3389/fpls.2023.1332428

**Published:** 2023-12-14

**Authors:** Rohit Bharati, Aayushi Gupta, Pavel Novy, Lucie Severová, Karel Šrédl, Jana Žiarovská, Eloy Fernández-Cusimamani

**Affiliations:** ^1^ Department of Crop Sciences and Agroforestry, The Faculty of Tropical AgriSciences, Czech University of Life Sciences Prague, Prague, Czechia; ^2^ Department of Botany and Plant Physiology, Faculty of Agrobiology, Food and Natural Resources, Czech University of Life Sciences Prague, Prague, Czechia; ^3^ Department of Food Science, Faculty of Agrobiology, Food and Natural Resources, Czech University of Life Sciences Prague, Prague, Czechia; ^4^ Department of Economic Theories, Faculty of Economics and Management, Czech University of Life Sciences Prague, Prague, Czechia; ^5^ Faculty of Agrobiology and Food Resources, Slovak University of Agriculture in Nitra, Nitra, Slovakia

**Keywords:** chromosome doubling, crop improvement, essential oil, *Melissa officinalis*, oryzalin, polyploid induction, polyploidization

## Abstract

*Melissa officinalis* L., a well-known herb with diverse industrial and ethnopharmacological properties. Although, there has been a significant lack in the breeding attempts of this invaluable herb. This study aimed to enhance the agronomical traits of *M. officinalis* through *in vitro* polyploidization. Nodal segments were micropropagated and subjected to oryzalin treatment at concentrations of 20, 40, and 60 mM for 24 and 48 hours. Flow cytometry, chromosome counting, and stomatal characteristics were employed to confirm the ploidy level of the surviving plants. The survival rate of the treated explants decreased exponentially with increasing oryzalin concentration and duration. The highest polyploid induction rate (8%) was achieved with 40 mM oryzalin treatment for 24 hours. The induced tetraploid plants exhibited vigorous growth, characterized by longer shoots, larger leaves, and a higher leaf count. Chlorophyll content and fluorescence parameters elucidated disparities in photosynthetic performance between diploid and tetraploid genotypes. Tetraploid plants demonstrated a 75% increase in average essential oil yield, attributed to the significantly larger size of peltate trichomes. Analysis of essential oil composition in diploid and tetraploid plants indicated the presence of three major components: geranial, neral, and citronellal. While citronellal remained consistent, geranial and neral increased by 11.06% and 9.49%, respectively, in the tetraploid population. This effective methodology, utilizing oryzalin as an anti-mitotic agent for polyploid induction in *M. officinalis*, resulted in a polyploid genotype with superior morpho-physiological traits. The polyploid lemon balm generated through this method has the potential to meet commercial demands and contribute significantly to the improvement of lemon balm cultivation.

## 1 Introduction

*Melissa officinalis*, commonly known as lemon balm, is a perennial crop belonging to the Lamiaceae family. It is widely cultivated for culinary uses, herbal tea production, and essential oil extraction owing to its aromatic leaves with a lemony scent and flavor (Dastmalchi et al., 2008; Shakeri et al., 2016). Additionally, lemon balm possesses numerous medicinal properties. For instance, it has been used to remedy conditions such as gastrointestinal disease, rheumatoid arthritis, or neurological disorders in traditional Asian medicines (Shakeri et al., 2016; Miraj et al., 2017). In folk medicines of several European countries, it is used for treating insomnia, sore throat, cough, nervousness, hepatic, and biliary ailments (Shakeri et al., 2016). Several recent studies have also demonstrated lemon balm to possess antioxidant, hypolipidemic, anti-cancer, anxiolytic, antimicrobial, and anti-inflammatory effects (Shakeri et al., 2016; Miraj et al., 2017). These pharmacological effects are primarily attributed to the various phytochemicals present in the essential oils of lemon balm. The essential oils of *M. officinalis* also contain several citral isomers, largely geranial, and neral, providing the plant with its characteristic lemon-scented aroma (Shakeri et al., 2016).

Plant breeding plays a pivotal role in enhancing the genetic diversity and adaptability of plants. However, in the case of *M. officinalis*, breeding attempts have been conspicuously scarce. This lack of attention provides an intriguing opportunity to delve into the unexplored capabilities of this crop. While traditional breeding approaches like hybridization, mass selection, and selective breeding have been successful, they are often time-consuming and cost-intensive (Salma et al., 2017). However, there are faster and cost-effective approaches like synthetic polyploidization for developing new genotypes with superior traits. Synthetic polyploidization, where anti-mitotic agents are employed to produce polyploid genotypes of an existing one, is gaining popularity among researchers for its numerous advantages (Dhooghe et al., 2011; Gantait and Mukherjee, 2021; Bharati et al., 2023b).

The polyploids generated through polyploidization often display superior agronomical traits and outperform their progenitor genotype (Niazian and Nalousi, 2020; Gantait and Mukherjee, 2021). For instance, leaf area increased significantly in *Thymus vulgaris* by 10.4% after polyploidization, whereas in *Anemone sylvestris*, a remarkable increase of 122.23% in the leaf length was observed (Zahumenická et al., 2018; Homaidan Shmeit et al., 2020). Owing to this potency of polyploidization, researchers have also successfully employed this technique to enhance the essential oil yields across various medicinal and aromatic plants. Some examples include an increased amount of essential oils in *Thymus vulgaris* (Homaidan Shmeit et al., 2020), *Lippia integrifolia* (Iannicelli et al., 2016), *Mentha × villosa* (Moetamedipoor et al., 2022), *Mentha spicata* (Bharati et al., 2023a), and *Zingiber officinale* Roscoe (Zhou et al., 2020). Induced polyploid plants often display higher chlorophyll contents with an enhanced photosynthetic capacity (Cao et al., 2018; García-García et al., 2020; Ulum et al., 2021).

Considering these developments, it could be hypothesized that polyploidization could be an excellent breeding approach to trait improvement in *M. officinalis*. Previously, colchicine has been predominantly used to induce polyploidy across a wide range of plant species due to its thermostability and effectiveness (Dhooghe et al., 2011; Eng and Ho, 2019). However, colchicine has multiple reported side effects, such as abnormal growth, sterility, and gene mutation, and it can lead to toxicity to humans (Dhooghe et al., 2011; Beranová et al., 2022). As an alternative, mitosis-inhibiting herbicides, including oryzalin, are becoming popular among researchers. Given that oryzalin has a higher affinity towards plant tubulin, it is more effective and provides better results when compared with colchicine at much lower concentrations (Ebrahimzadeh et al., 2018; Niazian and Nalousi, 2020). The application of anti-mitotic compounds to plants can be carried out either in *ex-vitro* or *in vitro* conditions. While *ex-vitro* conditions are more convenient, *in vitro* conditions offer better control and effectiveness, making them the preferred option for inducing polyploidy (Bharati et al., 2023a).

The objective of the study was to induce polyploidy in *M. officinalis* using oryzalin as an anti-mitotic agent under *in vitro* conditions. The current study also aims to assess the potential impact of polyploidization towards the enhancement of agronomical traits, including essential oil yield and anatomical, morphological, and photosynthetic attributes in lemon balm. The findings of this study will form a basis for breeding attempts in lemon balm and other medicinal and aromatic herbs. The developed protocol can be adopted at a commercial scale to generate novel genotypes with superior characteristics of various medicinal and aromatic plants.

## 2 Materials and methods

### 2.1 Procurement of plant materials and *in vitro* establishment

Plant samples of *M. Officinalis* (2n=2x=32) were obtained from the maintained medicinal plant collection at the botanical garden of the Faculty of Tropical Agrisciences, Czech University of Life Sciences Prague, Czech Republic. These samples were held in a greenhouse with an average temperature of 23°C and a relative humidity of 70-80%. Stems with a minimum of 5 nodes were carefully selected and washed with running tap water for 10 minutes. Nodal segments of approximately 1 cm were then cut and subjected to surface sterilization. The sterilization began by treating the explants with 70% ethanol (*v/v*) for 2 minutes. Subsequently, the explants were immersed and continuously stirred in a 1% (*v/v*) solution of sodium hypochlorite (commercial bleach-SAVO, NaClO) containing two drops of Tween-20 for 10 minutes. Afterward, the explants were rinsed three times using sterile double distilled water. The treated explants were then transferred to a laminar airflow hood for inoculation. The sterilized nodal segments were placed in 250 mL Erlenmeyer flasks containing approximately 50 mL of Murashige and Skoog’s basal medium (Murashige and Skoog, 1962). The medium was supplemented with 3% sucrose and 0.8% agar, and the pH was adjusted to 5.7 ± 0.1. The inoculated cultures were maintained in a growth room with a temperature of 24/20 ± 1°C and relative humidity between 65-70%. The photoperiod was set to 16/8 hours (light/dark) using white, fluorescent lamps with an intensity of 3800 lux (51.3 µmol m^−2^ s^−1^). The plants were subcultured every 14 days until a sufficient number of plants were obtained for polyploid induction.

### 2.2 Anti-mitotic agent treatment

Nodal segments of approximately 1-1.5 cm were placed in beakers containing MS medium and left for 48 hours before the treatments were applied. Oryzalin (Sigma-Aldrich, Prague, CZ) was dissolved in 1% Dimethyl sulfoxide (DMSO) to create three oryzalin solutions of different concentrations (20, 40, and 60 μM). These oryzalin solutions were then carefully poured onto the explants and maintained for 48 hours in a sterile laminar flow box until all the explants were submerged. The experiment consisted of six treatments labeled T1 to T6, which involved using oryzalin at different concentrations and exposure durations. The specific treatments were as follows: T1 (20 μM for 24 hours), T2 (20 μM for 48 hours), T3 (40 μM for 24 hours), T4 (40 μM for 48 hours), T5 (60 μM for 24 hours), and T6 (60 μM for 48 hours). A control variant (Control) was also used, where explants were subjected to MS medium without any oryzalin treatment. A total of 50 nodal segment explants were used for each treatment. After the treatment period, all the treated samples were taken out of the oryzalin solutions and rinsed three times with sterile double-distilled water. These washed samples were then placed back onto the MS medium and maintained for six months, with regular subculturing every 30 days. Every three months, the ploidy level of the samples was determined using a flow cytometer (Partec GmbH in Münster, Germany).

### 2.3 Transfer to the *ex-vitro* conditions

The control and the identified polyploid plants were transferred in a greenhouse with an average temperature of 23°C and relative humidity between 60-70%. The plants were subjected to primary and secondary hardening. Primary hardening mainly involved transferring the plants in a pot (5×5 cm) containing garden soil and perlite (3:1 ratio) under polythene covering for seven days. Following this, the polythene covering was removed, and the plants were subjected to an additional seven days under greenhouse conditions. Subsequently, the plants were transferred to field conditions (50°07’51.1”N 14°22’15.2”E) for a 60-day period, during which morphophysiological data and plant samples were collected for further analysis.

### 2.4 Morphological parameters

Morphological data were collected from the control diploid and induced polyploid plants to assess the influence of polyploidization. The evaluated parameters included: the number of shoots, shoot length, number of nodes per shoot, internodal distance, number of leaves per shoot, leaf area, leaf thickness, stem thickness, and wet weight. Data were also collected for floral and seed characteristics. A total of 20 observations were taken for all the attributes, excluding seed weight, which was determined by measuring the weight of 100 seeds across 10 replicates.

### 2.5 Stomatal observation

The length and width of stomata, along with the stomatal density from diploid and tetraploid plants, were compared using the nail polish impression method previously described (Grant and Vatnick, 2004; Parsons et al., 2019). The abaxial surface of the leaves was coated with clear nail polish and allowed to dry. Subsequently, imprints were obtained using transparent tape and placed on a glass slide to measure the size and density of stomata under a light microscope at 60× and 100× magnification.

### 2.6 Flowcytometry analysis

Flow cytometric analyses were performed as described by Beranová et al. (2022). Briefly, a leaf section of approximately 1cm^2^ was placed in a petri dish containing 1 mL of Otto I buffer (0.1 M C_6_H_8_O_7_, 0.5% Tween 20) and chopped using a razor blade. The crude suspension was then filtered through a nylon mesh (50 µm), and the filtrate was collected. After that, 1 mL of Otto II buffer (0.4 M Na_2_HPO_4_·12 H_2_O) was added to the filtrate containing 2 μg/mL of DAPI (fluorescent dye). The prepared samples were analyzed through a Partec PAS flow cytometer (Partec GmbH, Münster, DE), where a minimum of 10000 nuclei per sample were measured. The relative content of DNA was recorded in the form of a histogram using the Flomax software package (Version 2.3).

### 2.7 Chromosomes counting

Chromosome counting was performed following a previously published method (Bharati et al., 2023a) with slight adjustments. Approximately 1 cm long fresh root tips were collected in the early morning between 7 and 8 o’clock and immersed in a saturated solution of Paradichlorobenzene at room temperature for 2 hours. Subsequently, the root tips were rinsed three times with distilled water and submerged in a freshly prepared solution of ethyl alcohol and acetic acid (3:1) (*v/v*) for 60 minutes at room temperature. After removing the roots from the solution, they were washed thrice with distilled water before undergoing the hydrolysis and staining steps. The root tips were then subjected to a 10-minute incubation in a 1 N HCl solution at 60°C, followed by staining with Schiff’s reagent for 1 hour. Finally, the root tips were further trimmed to approximately 0.2 cm and placed on a glass slide with a drop of 2% orcein-acetic solution for visualization under a BX51 Olympus light microscope (Olympus Optical Co., Tokyo, Japan) at a magnification of 100×.

### 2.8 Non-destructive and destructive chlorophyll estimation

Non-destructive chlorophyll estimation was carried out using a SPAD-502, Minolta Camera CO, Japan, to compare the relative chlorophyll content between the diploid and tetraploid genotypes. A total of 20 measurements from each genotype were taken. To further validate the SPAD values, a previously established destructive method (Mosa et al., 2018) with slight modifications was used to estimate chlorophyll levels among the diploid and tetraploid genotypes. Fresh leaf samples were obtained from control diploid and induced tetraploid plants and were crushed into a fine powder using liquid nitrogen and a mortar and pestle. Accurately, 300 mg of the powdered sample was mixed with 5 mL of 80% acetone (*v/v*) and shaken in the dark for 15 minutes. Afterward, the mixture was centrifuged for 15 minutes at 4°C and 3000 rpm. The resulting liquid (supernatant) was collected and diluted with 80% acetone (*v/v*) in a 1:5 ratio. Finally, the absorbance of chlorophyll a (663 nm) and chlorophyll b (645 nm) was measured using a spectrophotometer. The total chlorophyll content in the diploid and tetraploid plants were calculated as previously described by Manzoor et al. (2018). Destructive chlorophyll estimation was carried out in three replicates.

### 2.9 ICP-MS analysis of micro and macronutrients

Approximately 0.2 g of dried samples from control and polyploid genotypes were measured and placed in a quartz vessel. Then, 2 mL of H_2_O_2_ (Rotipuran^®^, Carl Roth, Germany) and 4 mL of HNO_3_ (Analpure^®^, Analytika, Czech Republic) were added to the samples. The prepared samples were digested in a closed vessel microwave system at 180°C for 20 mins. After digestion, the solutions were transferred to 50 mL polypropylene tubes and filled with Milli-Q water (≥ 18.2 MΩ cm-1; MilliQ system, Millipore, SAS, France) to reach a final volume of 45 mL. The elemental concentration of certain macro and micro elements was then examined using inductively coupled plasma mass spectrometry (ICP-MS; Agilent 7700×, Agilent Technologies Inc., USA). To ensure quality, a certified reference material, specifically Peach leaves (SRM-1547, NIST), was used. For each genotype, three biological and three technical replicates were examined.

### 2.10 Comparison of chlorophyll fluorescence kinetics

To assess the photosynthetic performance of the diploid and tetraploid genotypes, we employed a portable FluorPen FP110 (manufactured by PSI, Czech Republic) and a MultispeQ V 2.0 device connected to the PhotosynQ platform (www.photosynq.org) with their default settings. These measurements were conducted on diploid and tetraploid plants under field conditions, with data collected at three different times of the day: morning (8:00 AM), afternoon (1:00 PM), and evening (5:00 PM). For FluorPen, the intact leaves were dark-adapted using leaf clips for 30 minutes. All the measured and calculated parameters from both the portable chlorophyll fluorometer included: LEF- linear Electron Flow, gH+ - proton conductivity of the thylakoid membrane, NPQt- Non-Photochemical Quenching, Phi2- Quantum Yield of Light-Adapted Photosystem II (PSII) Electron Transport, Fv/Fm -maximum quantum yield of PSII photochemistry, PIabs - absorption flux per active reaction center, ABS/RC - absorption per active reaction center. For the derivations and definitions of these parameters, one can refer to Stirbet and Govindjee (2011); Banks (2017); Kuhlgert et al. (2016).

### 2.11 Essential oil extraction and GC/MS analysis

Fresh aerial parts of diploid control and tetraploid plants were collected after 60 days of cultivation and dried until a constant dry weight was achieved in a drying oven (UF55plus, Memmert, USA) at 30°C. The dried samples were then subjected to hydro-distillation using a Clevenger-type apparatus in triplicate for two hours. The extracted oils were subsequently dried over anhydrous sodium sulfate and stored in airtight glass vials at 4°C until they were used for Gas chromatography-mass spectrometry (GC/MS) analysis. To identify the components of the essential oil, an Agilent 7890A gas chromatograph (GC) coupled with an Agilent 5975C single-quadrupole mass detector was utilized. The quantification was performed using an Agilent 6890A GC with a flame ionization detector (FID). Both instruments were equipped with an HP-5MS (30m x 250 μm x 0.25 μm) non-polar column (Agilent, Santa Clara, CA, USA). One µL of sample diluted 1:1000 in hexane (VWR, Stribrna Skalice, Czech Republic) was injected in the split ratio of 12:1 into the inlet heated to 250°C. The initial oven temperature was set to 60°C for 3 min, then it was gradually increased at the rate of 5°C/min up to 231°C and kept constant for 10 min. The FID detector was heated to 250°C. Helium was used as a carrier gas at the flow rate of 1 mL/min. The MS analysis was carried out in full scan mode with the electron ionization energy set to 70 eV. The relative percentage content was expressed as the ratio of individual peak area to the total area of all peaks. The identification of the essential oil components was based on a comparison of mass spectra and Kovat’s retention indices with those in the National Institute of Standards and Technology Library (NIST, USA) as well as in the literature (Adams, 2007). The identification of 9 components were confirmed using authentic standards. All the standards were obtained from Sigma–Aldrich (Prague, Czech Republic).

### 2.12 Statistical analysis

The data from the morphological, biochemical, and anatomical parameters of diploid and induced polyploid genotypes were subjected to statistical analysis using student’s t-test. All statistical tests were conducted with a significance level set at *p <* 0.05 using the Microsoft Excel 2021 software package. The graphs presented in the study were generated using GraphPad Prism (Version 9.4.0). Microscopic images were measured and analyzed using Image J software (IJ 1.46r).

## 3 Results

### 3.1 Effect of oryzalin on survival and polyploid induction rate

**Figure 1:**
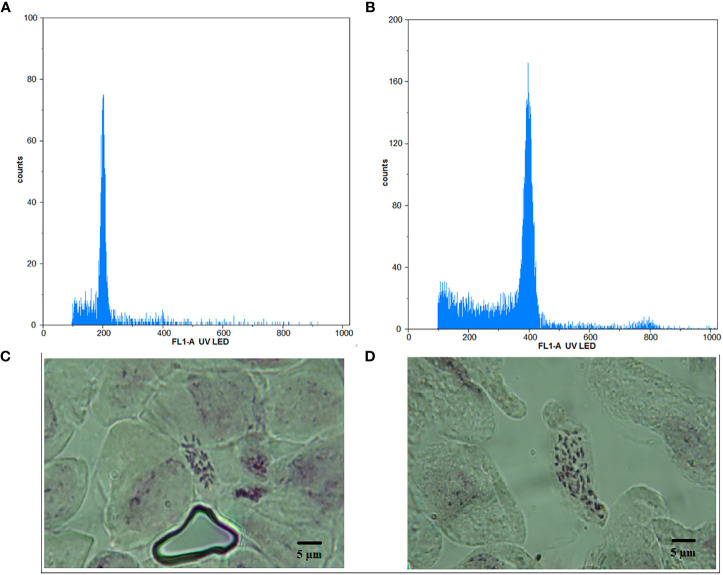
Histogram obtained from flowcytometry analysis for **(A)** diploid and **(B)** tetraploid plant, depicting relative DNA content along with chromosomes under 100x magnification for **(C)** diploid and **(D)** tetraploid plant. Bar = 5 μm.

The micropropagation of *M. officinalis* was found sufficient on the MS growth medium without any plant growth regulators. Out of the 300 nodal segments treated to various concentrations and duration of oryzalin, 113 plants survived. The survival rate of the plants decreased significantly with the increasing concentration of oryzalin (**Table 1**). The highest and the lowest survival rate were observed in T1 (54%) and T6 (24%), respectively (**Table 1**). The histograms generated through the flowcytometric analysis were effective in distinguishing the induced polyploids from the diploid (**Figures 1A, B**). A total of 8 polyploids were identified among the surviving population. The polyploid induction rate was highest in T3 (8%), while T4, T5, and T6 exhibited lower effectiveness, resulting in yields of just 4%, 2%, and 2%, respectively. The treatments T1 and T2 were found ineffective, with a polyploid induction rate of 0%. The ploidy of the control and polyploid genotypes were also validated through chromosome counting. It was affirmed that the chromosome numbers in the treated plants were doubled (2*n* = 4*x* = 64) compared with the diploid genotype (2*n* = 2*x* = 32) (**Figures 1C, D**).

**Table 1:** Polyploidization and survival rate of nodal segments of *M. officinalis* treated with oryzalin.

Treatment	Oryzalin (µM)	Number of treated explants	Time of treatment (h)	Survival rate (%)	Polyploid induction rate (%)
Control	0	50	n.a	100 ± 0.0	n.a
T1	40	50	24	54 ± 6.5^a^	0.0 ± 0.0
T2	40	50	48	44 ± 6.3^b^	0.0 ± 0.0
T3	60	50	24	36 ± 5.9^c^	8.00 ± 0.77^a^
T4	60	50	48	36 ± 5.9^c^	4.00 ± 0.42^b^
T5	80	50	24	32.5 ± 5.4^c^	2.00 ± 0.48^c^
T6	80	50	48	24 ± 6.5^d^	2.00 ± 0.48^c^

Different superscript letters within the same column differ significantly (Student’s t-test, p < 0.05%). ‘n.a’ indicates not applicable.

### 3.2 Morpho-physiological characteristics

**Figure 2:**
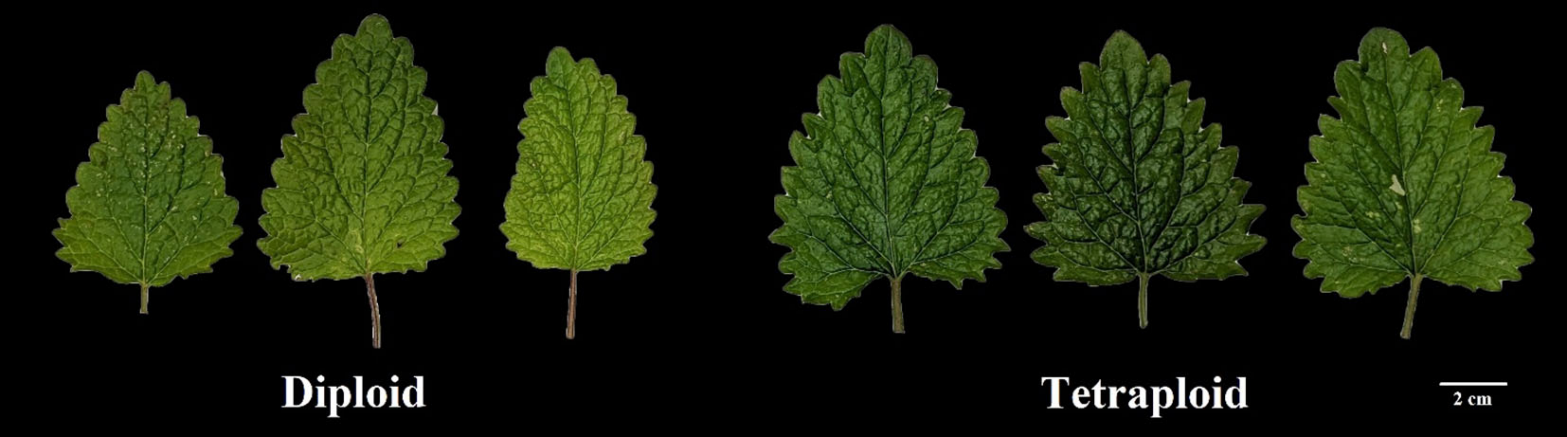
Comparison of leaf morphology from mother diploid and induced tetraploid plant.

**Figure 3:**
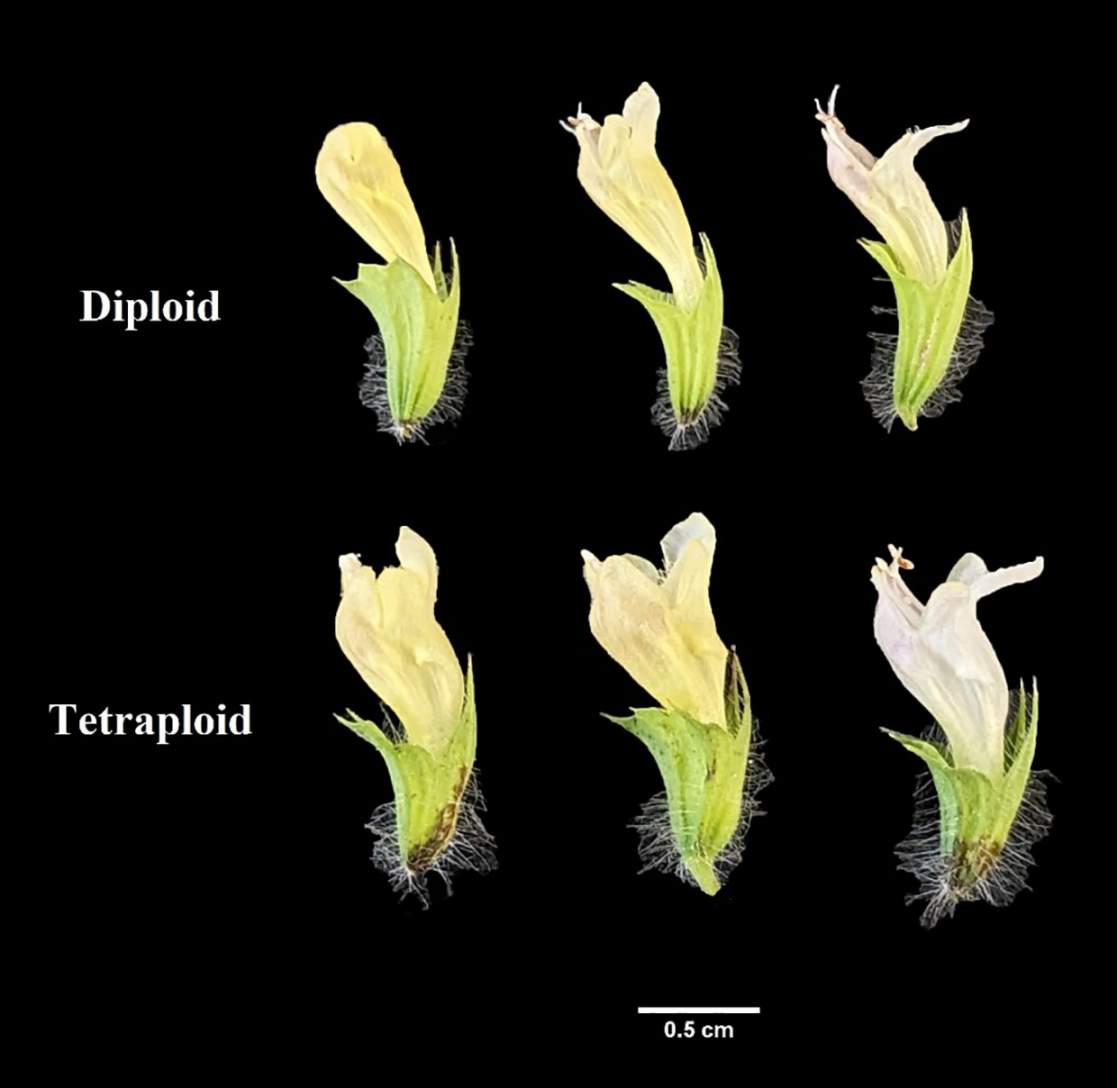
Comparison of floral characteristics between mother diploid and induced tetraploid plant.

**Figure 4:**
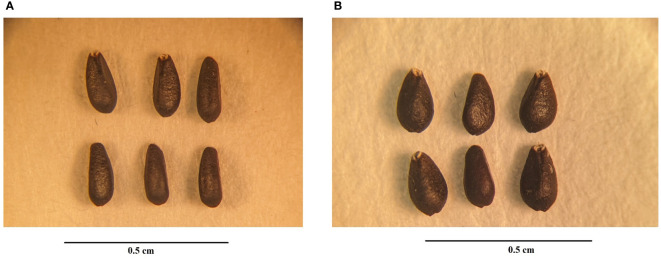
Morphology and structure comparison of seeds from **(A)** control diploid and **(B)** Induced tetraploid plants of *M. officinalis*.

Significant differences were observed in both the morphological and physiological characteristics of diploid and tetraploid plants outlined in **Table 2**. Tetraploid plants displayed a noteworthy increase in average shoot length and the number of nodes per shoot, with respective increments of 34.51% and 21.23% compared with the diploid control genotypes. Conversely, the average number of shoots per plant decreased in the tetraploid population, while internodal distance remained unaffected. Notably, leaf area, leaf thickness, and the number of leaves per shoot were positively impacted in tetraploid plants, exhibiting remarkable increases of 18.78%, 45.44%, and 52.63%, respectively, compared with the diploid plants (**Figure 2**; **Table 2**). Furthermore, a significant rise in average stem thickness was observed, elevating from 2.8 mm in diploid plants to 3.48 mm in tetraploid plants. The average fresh biomass per plant also experienced an upward trend, reaching 101.76 ± 7.82 g in tetraploids, as opposed to 85.3 ± 6.08 g in diploid plants (**Table 2**). Floral characteristics also displayed superior attributes in tetraploids. Specifically, the average length of the flower, width of the calyx, and width of the corolla exhibited an upward trend in tetraploid plants, with respective increases of 10.90%, 55.30%, and 30.04%. However, the length of the calyx remained unaffected (**Figure 3**; **Table 3**). The average seed weight exhibited a significant increase in tetraploid plants, reaching 108.6 ± 3.8 mg for every 100 seeds, compared to 73.15 ± 2.48 mg in diploid plants. Likewise, the average seed width in the tetraploid population expanded to 12.01 ± 1.04 mm from the 9.14 ± 0.62 mm observed in diploid plants. However, the average seed length did not display a significant difference (**Figure 4**; **Table 3**).

**Table 2:** Morpho-physiological characteristics of diploid control and tetraploid genotypes of *M. officinalis*.

Variant	Number of shoots	Shoot length (cm)	Number of nodes per shoot	Internodal distance (cm)	Number of leaves per shoot	Leaf area (cm^2^)	Leaf thickness (mm)	Stem thickness (mm)	Wet weight (g)
Diploid	24.28 ± 1.79^a^	19.96 ± 3.81^b^	5.98 ± 0.86^b^	4.11 ± 1.29^a^	11.98 ± 1.95^b^	26.54 ± 3.88^b^	0.38 ± 0.05^b^	2.8 ± 0.41^b^	85.3 ± 6.08^b^
Tetraploid	21.76 ± 2.07^b^	26.85 ± 1.24^a^	7.25 ± 1.02^a^	3.32 ± 0.63^a^	14.23 ± 1.84^a^	38.6 ± 2.41^a^	0.58 ± 0.08^a^	3.48 ± 0.53^a^	101.76 ± 7.82^a^

Different superscript letters within the same column differ significantly (Student’s t-test, p < 0.05%) (n=20).

**Table 3:** Flower and seed characteristics of diploid and induced tetraploid genotypes of *M. officinalis*.

Variant	Length of flower (mm)	Length of calyx (mm)	Width of calyx (mm)	Width of corolla (mm)	Average seed length (mm)	Average seed width (mm)	Average seed weight of 100 seeds (mg)
Diploid	13.57 ± 0.62^b^	7.18 ± 0.94^a^	3.49 ± 0.02^b^	4.76 ± 0.28^b^	19.05 ± 0.57^a^	9.14 ± 0.62^b^	73.18 ± 2.48^b^
Tetraploid	15.05 ± 0.46^a^	7.7 ± 1.21^a^	5.42 ± 1.22^a^	6.19 ± 0.32^a^	19.35 ± 0.53^a^	12.01 ± 1.04^a^	108.06 ± 3.8^b^

Different superscript letters within the same column differ significantly (Student’s t-test, p < 0.05%) (n=20).

#### 3.2.1 Anatomical comparison

**Figure 5:**
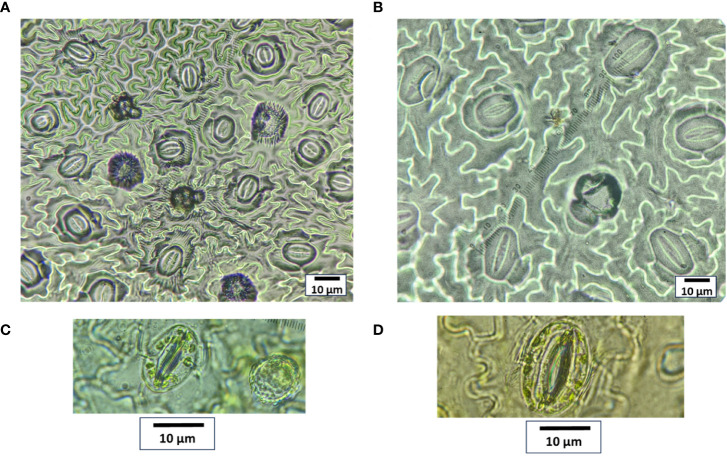
Stomata size and frequency comparison between **(A, C)** control diploid and **(B, D)** induced tetraploid plant. The images **(A, B)** were captured under 60× and **(C, D)** under 100× magnification.

**Figure 6:**
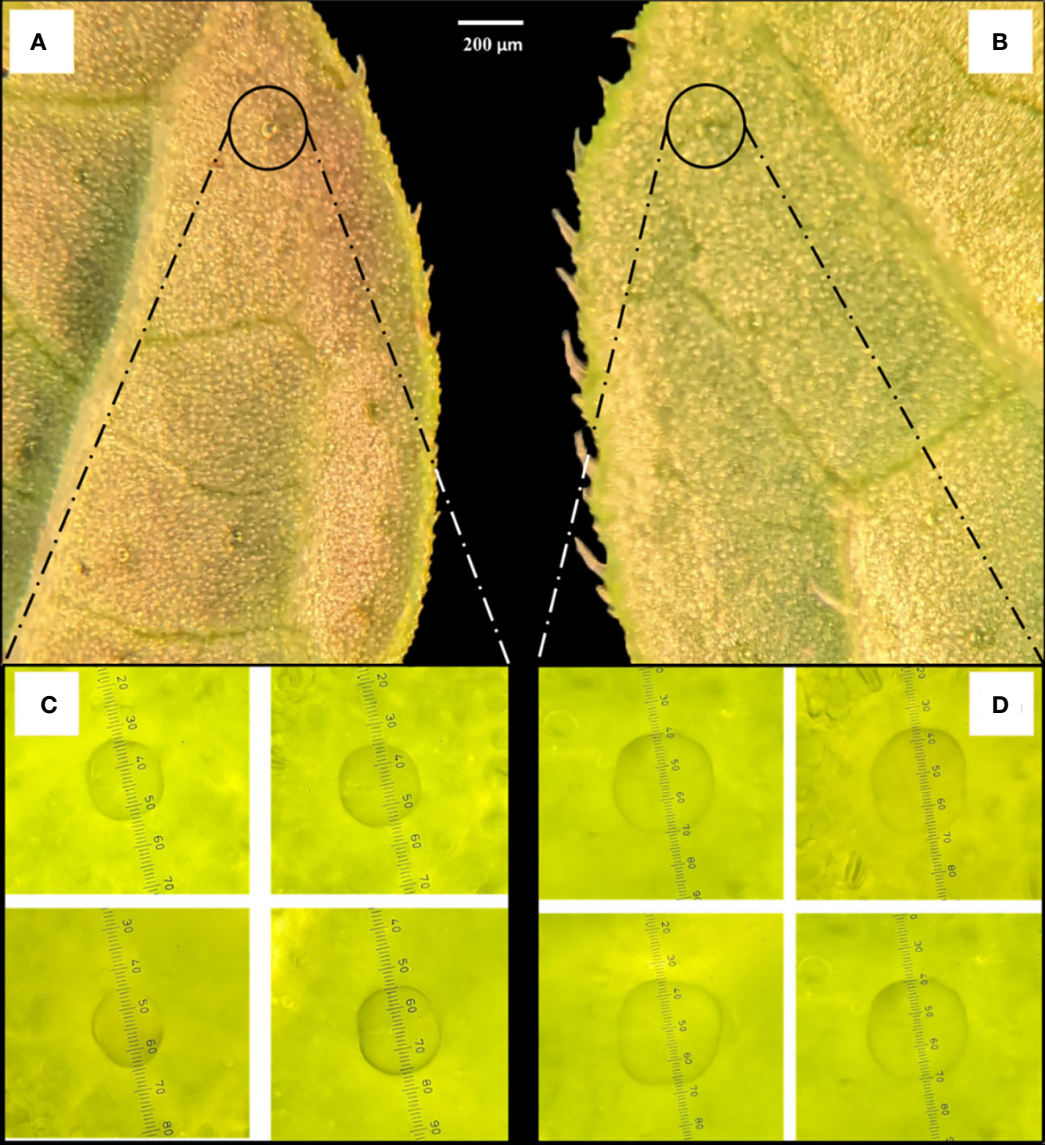
Comparison of peltate trichome gland sizes between **(A, C)** diploid and **(B, D)** induced tetraploid plants. The images were captured under 10× magnification **(A, B)** and 40× magnification **(C, D)**. The measurements in C and D are presented in micrometers (µm).

Notable differences in the anatomical characteristics between the tetraploid and diploid genotypes were observed. The stomatal frequency decreased significantly in the tetraploid population, although the average length and width of the stomata increased from 11.34 ± 1.95 µm and 7.86 ± 1.05 µm in diploid to 20.76 ± 1.52 µm and 12.57 ± 0.79 µm in tetraploid plans, respectively (**Table 4**; **Figures 5A-D**). Furthermore, the length of the guard cells were positively influenced and increased from 16.21 ± 2.34 in diploid to 25.72 ± 2.23 µm tetraploid plants. Additionally, the peltate trichomes density, which is chiefly responsible for the oil secretion, was also influenced, and the average diameter of oil-secreting glands increased from 20.85 ± 1.61 μm in diploid to 30.24 ± 2.18 in the tetraploid population (**Figures 6A-D**).

**Table 4:** Comparison of stomatal characteristics between diploid and induced polyploid genotypes of *M. officinalis*.

Variant	Average number of stomata per magnification field (60x)	Average stomata length (µm)	Average stomata width (µm)	Average guard cell length (µm)
Diploid	24.33 ± 3.21^a^	11.34 ± 1.95^b^	7.86 ± 1.05^b^	16.21 ± 2.34^b^
Tetraploid	10.00 ± 1.50^b^	20.76 ± 1.52^a^	12.57 ± 0.79^a^	25.72 ± 2.23^a^

Different superscript letters within the same column differ significantly (Student’s t-test, p < 0.05%) (n=20).

#### 3.2.2 Chlorophyll content

**Figure 7:**
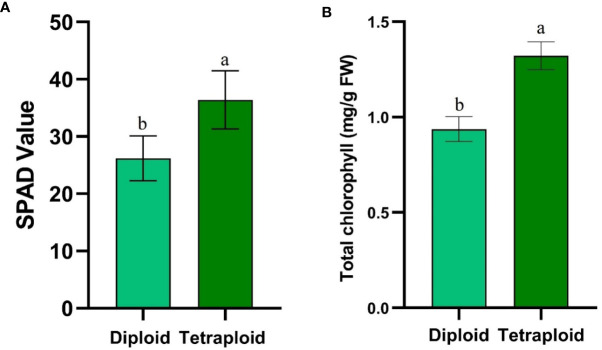
Graphs depicting **(A)** SPAD values and **(B)** Total chlorophyll content comparison between control diploid and induced tetraploid plant. Different superscript letters on the vertical bars differ significantly (*p* < 0.05).

The average SPAD values increased significantly rising from 26.20 ± 3.91 in diploid to 36.39 ± 5.07 in tetraploid plants (**Figure 7A**). Similarly, destructive estimation of chlorophyll content revealed a significant increase from 0.93 ± 0.05 mg/g FW in diploid plants to 1.32 ± 0.07 mg/g FW in tetraploid plants (**Figure 7B**).

### 3.3 Chlorophyll fluorescence kinetics

**Figure 8:**
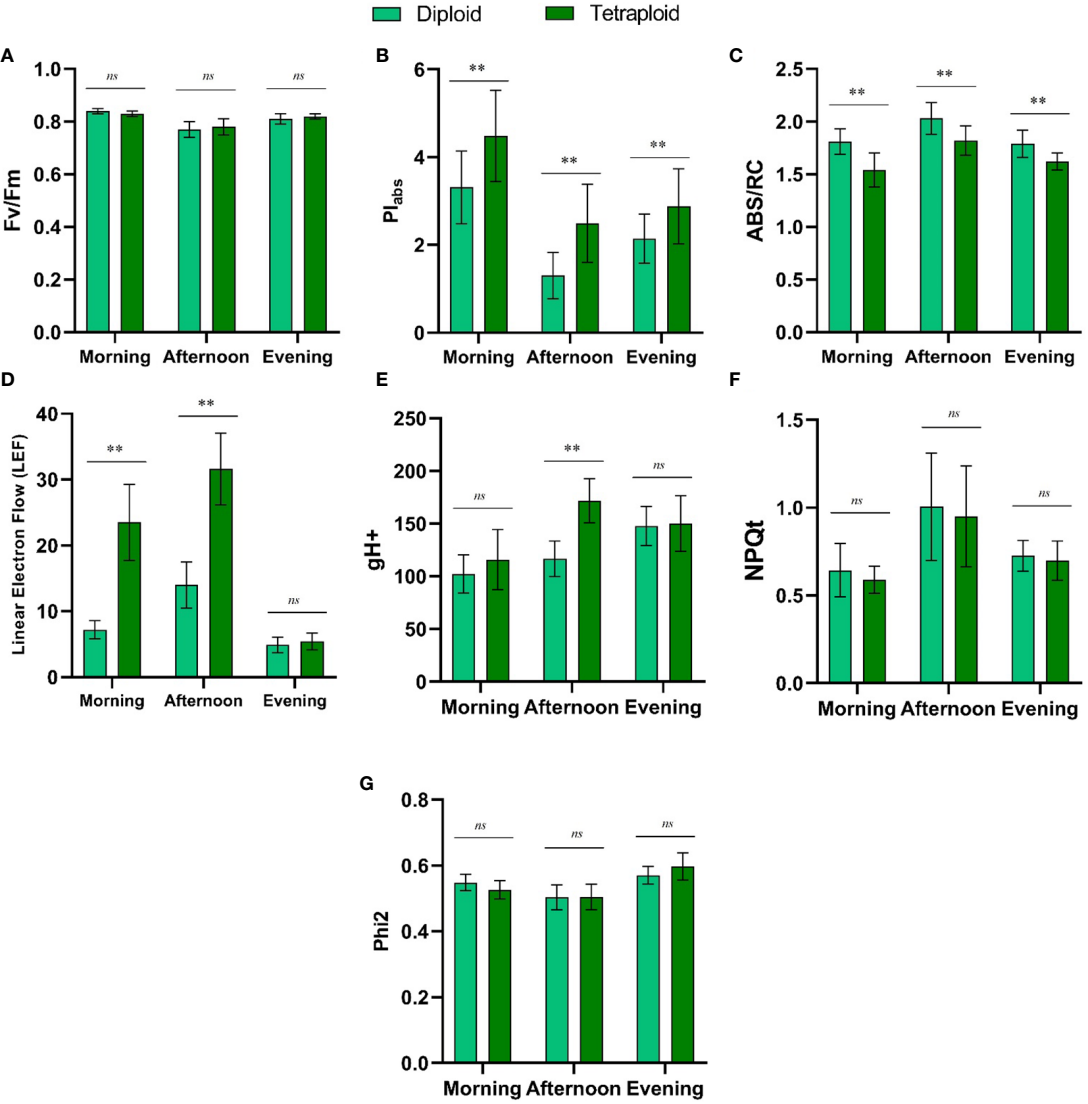
Comparison of Diurnal Fluctuations in Chlorophyll Fluorescence Metrics **(A)** Fv/Fm, **(B)** PIabs, **(C)** ABS/RC, **(D)** Linear Electron Flow (LEF), **(E)** gH+, **(F)** NPQt, and **(G)** Phi2 between Control Diploid and Induced Tetraploid Plants. ‘**’ Signifies Significant Differences, while ‘*ns*’ Indicates No Significant Difference at p<0.005.

In our comparative study of diploid and induced tetraploid genotypes of *M. officinalis*, we observed that Fv/Fm, NPQt, and Phi2 values exhibited no significant differences between the two genotypes throughout the day (**Figures 8A, F, G**). Conversely, PIabs and ABS/RC values displayed consistent and statistically significant distinctions between diploid and tetraploid plants across all time points (morning, afternoon, and evening) (**Figures 8B, C**). For PIabs, diploid values ranged from 1.3 to 3.31, while tetraploid values were consistently higher, ranging from 2.88 to 4.48. ABS/RC values also exhibited significant differences, reflecting variations in absorption per active reaction center between the two genotypes. In the diploid genotype, ABS/RC values ranged from 1.79 to 2.03, whereas in the tetraploid genotype, values ranged from 1.54 to 1.82. Linear Electron Flow (LEF) showed a significant increase in the tetraploid genotype in the morning and afternoon but became statistically insignificant in the evening. In the morning, LEF was 7.19 for diploid and 23.52 for tetraploid, while in the afternoon, it was 14.0 for diploid and 31.635 for tetraploid (**Figure 8D**). Proton conductivity of the thylakoid membrane (gH+) displayed a significant difference between the genotypes only in the afternoon, with values of 116.71 for diploid and 171.82 for tetraploid (**Figure 8E**). These results underscore the genotype-specific variations in photosynthetic parameters, particularly PIabs and ABS/RC, and their dependency on the time of day, suggesting intricate regulatory mechanisms at play in response to changing environmental conditions.

### 3.4 Macro- and micronutrients content

The results of the ICP-MS analysis comparing diploid and tetraploid genotypes of *M. officinalis* revealed distinct changes in the elemental composition. The potassium concentration increased significantly while the calcium concentrations decreased in the induced polyploids. Phosphorus, ferrous, and boron concentrations were unaffected by chromosome doubling and remained same between the two genotypes. On the other hand, copper, manganese, and zinc concentrations were observed to decrease in the tetraploid genotype compared with the diploid genotype (**Table 5**).

**Table 5:** Macronutrient and micronutrient analyzed from leaves of diploid and induced polyploids of *M. officinalis* plants (mg/kg).

Variant	K	Ca	P	Fe	Cu	Zn	B	Mg
Diploid	27867.99±911.83^b^	11755.70±302.79^a^	4159.51±20.59^a^	120.69±10.78^a^	14.53±0.33^a^	49.09±3.42^a^	23.87±0.82^a^	5294.86±54.97^a^
Tetraploid	34438.93±249.30^a^	8019.07±441.131^b^	3651.71±227.62^a^	100.71±6.07^a^	11.73±0.61^b^	31.50±2.75^b^	24.14±0.84^a^	4450.96±134.95^b^

Different superscript letters within the same column differ significantly (Student’s t-test, p < 0.05) (n=20).

### 3.5 Essential oil yield and composition

The essential oil yields were 0.21% (*v/w*) in tetraploid plants, whereas the diploid plants yielded only 0.12% (*v/w*) of essential oil. A total of 21 components were identified, which represents 90.97% and 92.49% of the total composition of the oils from diploids and tetraploid, respectively (**Table 5**). The major components were geranial, neral, and citronellal, constituting 73.23% and 80.10% of the total composition in diploids and tetraploids, respectively. The remaining components didn´t exceed 5%. Although, two of the significant minor components, geranyl acetate and caryophyllene oxide reduced and were 4.11 and 4.23% in diploid and 3.42 & 2.68% in tetraploid, respectively. Among the major components, neral and geranial increased significantly, rising from 27.60% and 30.22% in diploid to 39.23% and 43.57% in the tetraploid genotype, respectively. On the other hand, the other major component, citronellal, was unaffected by chromosome doubling (**Table 6**).

**Table 6:** Composition of diploid and tetraploid *Melissa officinalis* essential oils.

*RI* ^a^	*RI*_(*lit*)_^b^	Compound	Content (%)±SD ^c^				Identification^d^
			Diploid		Tetraploid		
700	700	Heptane	0.21±0.008		0.07±0.002		MS, RI, Std
800	800	Hexanal	0.32±0.004		0.08±0.002		MS, RI
852	854	*E*-2-Hexenal	0.44±0.001		0.32±0.001		MS, RI
980	980	β-Pinene	0.87±0.003		0.47±0.001		MS, RI, Std
1057	1055	Benzeneacetaldehyde	0.50±0.012		0.34±0.004		MS, RI
1099	1098	Linalool	0.35±0.010		0.51±0.001		MS, RI, Std
1147	1147	7-Methyl-3-methyleneoct-6-enal	0.48±0.006		0.39±0.008		MS, RI
1156	1153	**Citronellal**	**6.40±0.013**		**6.31±0.012**		MS, RI, Std
1167	1165	Isoneral	0.20±0.006		0.45±0.006		MS, RI
1184	1184	3,7-Dimethyl-3,6-octadienal	1.03±0.026		0.72±0.010		MS, RI
1244	1240	**Neral**	**27.60±0.017**		**30.22±0.016**		MS, RI, Std
1257	1255	Geraniol	0.06±0.002		0.85±0.002		MS, RI, Std
1264	1261	Methyl citronellate	1.29±0.004		0.51±0.001		MS, RI
1273	1270	**Geranial**	**39.23±0.040**		**43.57±0.022**		MS, RI, Std
1325	1323	Methyl geraniate	0.70±0.002		0.31±0.003		MS, RI
1385	1383	Geranyl acetate	4.11±0.007		3.42±0.003		MS, RI
1419	1418	β-Caryophyllene	0.00±0.000		0.17±0.005		MS, RI, Std
1430	1436	α-Bergamotene	0.27±0.003		0.22±0.002		MS, RI
1490	1493	β-Ionone	1.75±0.016		0.38±0.001		MS, RI
1580	1574	Germacrene D-4-ol	0.93±0.023		0.49±0.011		MS, RI
1582	1581	Caryophyllene oxide	4.23±0.021		2.68±0.004		MS, RI, Std
		Total identified	90.97		92.49		

a: Kovat´s retention indices measured on HP-5MS column; b: retention indices from literature; c: relative percentage content based on total area of all peaks (average of three extractions); d: Identification based on comparison of mass spectra (MS) and retention indices (RI) with NIST library and literature; Std: identification confirmed by co-injection with authentic standard.

The bold values represent the major components present in the essential oil of both the genotypes.

## 4 Discussion

Polyploidization attempts have been made in lemon balm using colchicine to obtain polyploid genotypes (Borgheei et al., 2010; Talei and Fotokian, 2020). Although, the previous studies failed to provide specific information on the concentration and duration effective for the induction of tetraploid plants in *M. officinalis.* On the other hand, oryzalin has never been assessed as an anti-mitotic agent in this species. In the current study, the use of oryzalin as an anti-mitotic agent successfully generated polyploids in *M. officinalis*, establishing the potency of oryzalin as an anti-mitotic agent in this plant species. The efficacy of oryzalin as an anti-mitotic agent has been well established. Oryzalin exhibits a higher affinity towards plant tubulin and lower toxicity than colchicine, making it a preferred choice for chromosome doubling in plants (Dhooghe et al., 2011; Niazian and Nalousi, 2020). For instance, a study by Sakhanokho et al. (2009), tested the efficacy of colchicine and oryzalin for inducing polyploids in Ornamental Ginger (*Hedychium muluense*), and oryzalin was found more effective with 15% induction frequency compared with colchicine with just 13%. Interestingly, the effective concentration for oryzalin was just 60 µM, whereas colchicine was 2.5 mM. Similar results where oryzalin performed better than colchicine have been reported in *Passiflora edulis* Sims. (Rêgo et al., 2011), *Watsonia lepida* (Ascough et al., 2008) and some of the *Vaccinium species* (Tsuda et al., 2013). It is worth noting that none of the treatments in our study resulted in mixoploidy, a phenomenon frequently observed in synthetic polyploidization. This suggests that *M. officinalis* may be inherently unstable in a mixoploid state and prefers to exist either as a diploid or a tetraploid when induced using oryzalin.

Morphological variations in the induced polyploid are a well-known phenomenon called the “gigas effect”. The induced polyploids often exhibit enlarged organs compared with their progenitors. For instance, a significant increase in leaf area, width, and thickness was observed in the induced polyploids of *Thymus vulgaris* and *Agastache foeniculum* L. (Talebi et al., 2017; Homaidan Shmeit et al., 2020). Similarly, a recent study on polyploidization in *Mentha spicata* elucidated that the polyploids displayed vigorous growth with larger organs. Specifically, the polyploids of *M. spicata* observed an increase in leaf area, leaf, and stem thickness and nearly doubled compared with the control genotype (Bharati et al., 2023a). Comparable observations were observed in the current study where the tetraploid plants exhibited a more robust growth compared with the diploid plants, characterized by longer shoots, a higher number of nodes per shoot, larger leaves, and an increased leaf count per shoot. As a result, the tetraploid plants exhibited a notably bushier growth habit. Comparing the floral characteristics, it was evident that the flower size increased significantly in the tetraploid compared with the diploid plants. Although, the floral traits were retained, such as petal number and flower color. Polyploidy-induced alterations in developmental processes may have influenced flower growth and differentiation, leading to changes in floral morphology. A similar increase in the size of the flower post-polyploidization has been reported across different plant species (Zahumenická et al., 2018; Bhattarai et al., 2021). The seed characteristics were influenced significantly where the tetraploid plants generated heavier and larger seeds. Similar increases in the seed size and weight in the polyploid genotypes have been reported in other plant species (Sadat Noori et al., 2017; Ding et al., 2023). The seed viability and the ploidy stability over generations need to be assessed and should be part of future studies.

Anatomical comparisons are frequently used as an indirect approach to assess the impact of polyploidization in plants. Stomatal observations were made to assess the difference between the stomata of the diploid and the induced tetraploid plants. The findings from the current study suggest that polyploidization affects the stomatal size significantly in this species, where it increased the length and width of the stomata by 83.06% and 59.92%, respectively, in the tetraploid population. A similar significant increase in stomatal size has been reported in lemon balm and other plant species from the Lamiaceae family due to chromosome doubling (Talei and Fotokian, 2020; Moetamedipoor et al., 2022; Bharati et al., 2023a). Although, with an increase in stomatal size, the density is often reported to decrease after polyploidization, which is consistent in the current study. The reduction in stomatal frequency is frequently linked to an increase in leaf epidermal cells, stomatal cells and a decrease in stomatal differentiation in induced polyploid plants (Shariat and Sefidkon, 2021).

Lemon balm subjected to polyploidization resulted in significantly higher chlorophyll content and similar increase in chlorophyll content in the induced polyploid plants is a well reported phenomenon (Feng et al., 2017; Münzbergová and Haisel, 2019; García-García et al., 2020). Further, the diurnal comparison of the chlorophyll fluorescence parameters provided valuable insights into the photosynthetic performance of the newly developed genotype across different time points of the day. Fv/Fm is a commonly used indicator of the maximum quantum yield of photosystem II (PSII), while NPQt and Phi2 are related to non-photochemical quenching and photosystem II quantum yield, respectively. These photosynthetic parameters showed no significant differences between the two genotypes throughout the day, indicating similar photosystem efficiency and photoprotective mechanisms (Stirbet and Govindjee, 2011), (Banks, 2017), and (Kuhlgert et al., 2016). However, PIabs and ABS/RC values consistently differed, with the tetraploid genotype exhibiting higher PIabs and lower ABS/RC, indicating that the tetraploid plants are more efficient in absorbing and utilizing light energy for photosynthesis (Kumar et al., 2020). Linear Electron Flow (LEF) was significantly higher in the tetraploid genotype in the morning and afternoon but leveled off in the evening. The tetraploid genotype’s enhanced LEF in the morning and afternoon may indicate a more efficient conversion of absorbed light energy into electron transport, possibly due to higher pigment content or improved energy distribution within the thylakoid membranes (Kuhlgert et al., 2016). Proton conductivity (gH+) showed differences only in the afternoon, with higher values in the tetraploid genotype. This result implies that the tetraploid plants may have an enhanced capacity for proton transport across the thylakoid membrane during periods of increased photosynthetic activity, which could contribute to their improved photosynthetic performance in the afternoon (Avenson et al., 2005).

Trichomes are vital structures found in lemon balm, primarily responsible for secreting essential oils. Surprisingly, previous research on polyploidization in *M. officinalis* did not assess its impact on these oil-secreting glands known as peltate trichomes. Furthermore, the authors did not evaluate the essential oil yield in the induced polyploid plants (Borgheei et al., 2010; Talei and Fotokian, 2020). Conversely, the current study revealed a significant 45% increase in the size of these oil glands in tetraploid plants compared with diploid plants, indicating a corresponding enhancement in overall essential oil production in the tetraploid variants. Correspondingly, the essential oil extraction through hydro-distillation revealed a 75% increase in the tetraploid plants compared with the diploid plants. Enhanced levels of essential oils induced through polyploidization have been reported in various medicinal and aromatic plants from the Lamiaceae family. For instance, polyploidization in *Mentha×villosa* and *Mentha spicata* recorded essential oils increase of 64% and 48.85% in the induced polyploid population, respectively (Moetamedipoor et al., 2022; Bharati et al., 2023a). A similar rise in the essential oil of 46% in the induced tetraploid plants was recorded in *Thymus vulgaris* (Homaidan Shmeit et al., 2020). The surge in essential oil production holds the potential to drive enhanced profitability across diverse sectors, including fragrance, cosmetics, aromatherapy, and herbal medicine, which heavily rely on the utilization of lemon balm-derived essential oils.

Determining the nutrient profile in the newly developed genotype of lemon balm holds important significance. Understanding how induced polyploidy affects nutrient composition and uptake provides valuable insights for optimizing cultivation practices to enhance the medicinal quality of the herb. This is particularly pertinent for medicinal plants where the concentration of bioactive compounds is often linked to nutritional status (Yadegari, 2017; Radha et al., 2021). Furthermore, understanding the nutrient profile of the new genotype with medicinal potential is imperative from a safety perspective. This is because the plant’s uptake of both essential and potentially toxic elements is crucial for discerning potential health risks associated with the consumption of these novel medicinal plants of lemon balm (De Souza et al., 2021). In the current study, the tetraploid genotype exhibited a noteworthy increase of 23.57% in potassium content compared with the control diploid genotype. However, no significant changes were observed in other macro and micro-nutrients, which remained unaffected or displayed significant decreases. An increase in the potassium concentration in the induced polyploid population has been previously reported in *Callisia fragrans* (Beranová et al., 2022). In the same study, polyploid genotypes with decreased calcium levels were also observed. It has been previously reported that the polyploidization can influence the metabolic pathways involved in nutrient uptake, transport, and accumulation, which could cause these disparities in the mineral concentration (Tossi et al., 2022).

GC-MS analysis revealed that diploid and tetraploid essential oils had 3 major components: neral, geranial, and citronellal, where neral and geranial increased by 9.49% and 11.06% in the tetraploid plants. Geranial and neral are predominantly present in a well-known monoterpene aldehyde known as citral (3,7-dimethyl-2,6-octadienal) (CTR), mainly found in citrus fruits and herbs. The CTR possesses various beneficial properties, including anti-cancer, antimicrobial, anti-inflammatory, spasmolytic, analgesic, and chemopreventive activities (Bakkali et al., 2008; Liao et al., 2015; Aprotosoaie et al., 2019; Silva et al., 2022). The increased presence of these compounds in the essential oil of tetraploid *M. officinalis* suggests that the newly obtained genotype through polyploidization might potentially display improved therapeutic effectiveness. However, this emphasizes the importance of further systematic research to explore the pharmacological potential of the essential oil produced by tetraploid plants.

## 5 Conclusions

In the present investigation, oryzalin has been identified as a potent anti-mitotic agent within the context of *M. officinalis*. Notably, the treatment with oryzalin successfully induced a tetraploid genotype (2 *n* = 4× = 64) from nodal segments of diploid plants (2*n* = 2*×* = 32). The tetraploid genotype exhibited normal growth and maintained a stable ploidy level consistently over time. The tetraploid genotypes displayed significantly thick, large, high number of leaves per plant with significantly larger stomata, higher chlorophyll content and higher photosynthetic performance. Furthermore, the tetraploid genotypes also exhibited a noteworthy elevation in essential oil content in *M. officinalis*. This synthetic polyploidization process resulted in an increase not only in overall essential oil yield but also in the concentrations of key essential oil components. The obtained genotype in the current study possesses high economic value due to its superior agronomical traits. The obtained genotype can be scaled up for commercial adoption to meet the rising demand and address the limited production, thereby generating significant economic benefits. Chromosome doubling may play a pivotal role in breeding *M. officinalis* and other medicinal and aromatics plants.

## Data availability statement

The original contributions presented in the study are included in the article/supplementary material. Further inquiries can be directed to the corresponding authors.

## Author contributions

RB: Conceptualization, Data curation, Investigation, Methodology, Software, Writing – original draft, Writing – review & editing. AG: Data curation, Investigation, Methodology, Writing – original draft, Writing – review & editing. PN: Formal Analysis, Investigation, Methodology, Writing – original draft, Writing – review & editing. LS: Formal Analysis, Funding acquisition, Resources, Writing – review & editing. KŠ: Formal Analysis, Funding acquisition, Resources, Writing – review & editing. JŽ: Formal Analysis, Resources, Writing – review & editing. EF-C: Conceptualization, Formal Analysis, Funding acquisition, Project administration, Supervision, Writing – review & editing.
